# Trust, autonomy, and informed consent: a qualitative investigation of youth experiences accessing contraception in Canada

**DOI:** 10.1080/26410397.2026.2678063

**Published:** 2026-06-01

**Authors:** Sarah Munro, Madeleine Ennis, Kaiya Jacob, Bimbola Olure, Zeba Khan, Hajar Seiyad, Ayla Arhinson, Ruby Abramson, Victoria Paller, Andrea Carson, Andrea J. Hoopes, Wendy V. Norman, Giuseppina Di Meglio

**Affiliations:** a Assistant Professor, Health Systems and Population Health, School of Public Health, University of Washington, Seattle, WA, USA; Affiliate Assistant Professor, Department of Obstetrics and Gynaecology, Faculty of Medicine, University of British Columbia, Vancouver, Canada.; b Research Associate, Department of Obstetrics and Gynaecology, Faculty of Medicine, University of British Columbia, Vancouver, Canada; c Research Coordinator, Department of Obstetrics and Gynaecology, Faculty of Medicine, University of British Columbia, Vancouver, Canada; d Research Coordinator, Department of Obstetrics and Gynaecology, Faculty of Medicine, University of British Columbia, Vancouver, Canada; e Research Assistant, Department of Obstetrics and Gynaecology, Faculty of Medicine, University of British Columbia, Vancouver, Canada; f Youth partner, Toronto, Canada; g Youth partner, Toronto, Canada; h Youth partner, Toronto, Canada; i Research Assistant, Department of Obstetrics and Gynaecology, Faculty of Medicine, University of British Columbia, Vancouver, Canada; j Project Consultant, London Health Sciences Centre, London, Canada; k Assistant Investigator, Kaiser Permanente Washington Health Research Institute, Seattle, WA, USA; l Professor, Department of Family Practice, Faculty of Medicine, University of British Columbia, Vancouver, Canada; m Associate Professor, Faculty of Medicine, Department of Pediatrics, McGill University, Montreal, Canada

**Keywords:** contraception, adolescent, informed consent, Canada, reproductive health services, qualitative research

## Abstract

We sought to understand, “*What are the contraception access experiences, beliefs, attitudes, knowledge and needs of youth in Canada, from the perspectives of youth and youth service providers?*” We explore person-centred contraception care and highlight the importance of fostering trust, autonomy, and informed consent. We conducted one-on-one semi-structured interviews with youth (aged 15–25) and healthcare providers (HCPs) across Canada, representing a diversity of rural and urban settings, gender, age, and ethnicities. Using social constructivist grounded theory, we identified key themes related to reproductive autonomy, trust, and informed consent. We analysed data from 79 youth and 27 HCPs, identifying five themes: (1) *Valuing shared decision making*; (2) “*Pushing*” *contraception: putting a stop to paternalism*; (3) *Seeking anti-oppressive care*; (4) *Contraception care is all about trust*; (5) *Practising control over my own body.* Participants valued shared decision-making and wanted to feel that “we’re in this together”. Both youth and HCP participants shared stories of HCPs “pushing contraception” on youth and experiences of medical discrimination which impeded feelings of safety and autonomy. In response, youth advocated for *anti-oppressive care* and identified the importance of “therapeutic relationship building,” emphasising that fostering safety with HCPs is “all about trust.” Finally, youth reported practising advocating for their reproductive autonomy to have control over their own body. Our research highlights the importance of shared decision-making, informed consent, and anti-oppressive care in fostering supportive and safe contraception access for youth. Results have implications for jurisdictions where youth face increasing barriers to contraception access. *DOI: 10.1080/26410397.2026.2678063*

## Plain language summary

Young people in Canada face more challenges than adults when trying to access contraception (birth control). However, their access experiences are not well understood. We aimed to understand the contraception access experiences and needs of young people in Canada. We interviewed young people and clinicians across Canada. We analysed these interviews to identify key themes related to autonomy, trust, and informed consent. We found that young people wanted to feel they were “in this together” with health workers when they discussed contraception. Young people and clinicians shared stories of clinicians “pushing contraception” on youth. They also shared experiences of youth being pressured to start and continue methods. Young people wanted equitable, respectful, and supportive care. They appreciated building relationships with health workers and said that feeling safe is “all about trust.” Finally, young people reported practising speaking up for themselves when making contraception choices. Our research suggests that trusted, respectful relationships help make young people feel supported and safe when making contraception choices.

## Résumé

Nous avons cherché à comprendre quelles sont les expériences, les croyances, les attitudes, les connaissances et les besoins des jeunes au Canada en matière d’accès à la contraception, du point de vue des jeunes et des prestataires de services pour la jeunesse. Nous avons étudié les soins de contraception centrés sur la personne et souligné combien il est important de favoriser la confiance, l’autonomie et le consentement éclairé. Nous avons mené des entretiens semi-structurés individuels avec des jeunes (âgés de 15 à 25 ans) et des professionnels de santé dans l’ensemble du Canada, représentant une variété de milieux ruraux et urbains, de genres, d’âges et d’origines ethniques. À l’aide de la théorisation enracinée constructiviste sociale, nous avons identifié des thèmes majeurs relatifs à l’autonomie reproductive, à la confiance et au consentement éclairé. Nous avons analysé les données de 79 jeunes et de 27 professionnels de santé, ce qui a permis d’identifier cinq thèmes: (1) accorder de l’importance à la prise de décision partagée; (2) « pousser » à la contraception: mettre un terme au paternalisme; (3) rechercher des soins anti-oppressifs; (4) comprendre que les soins en matière de contraception sont avant tout fondés sur la confiance; (5) assurer la maîtrise de son propre corps. Les participants valorisaient la prise de décision partagée et souhaitaient sentir qu’ils étaient tous concernés. Les jeunes et les professionnels participants ont partagé des témoignages sur des professionnels de santé qui « poussaient » les jeunes à la contraception et des expériences de discrimination médicale qui ont nui à leur sentiment de sécurité et d’autonomie. En réaction, les jeunes ont plaidé pour des soins anti-oppressifs et ont cité l’importance de l’établissement d’une relation thérapeutique, insistant sur le fait que la confiance est essentielle pour promouvoir un sentiment de sécurité avec les professionnels de santé. Enfin, les jeunes ont indiqué qu’ils s’exerçaient à défendre leur autonomie reproductive afin d’avoir la maîtrise de leur propre corps. Notre recherche met en lumière l’importance de la prise de décision partagée, du consentement éclairé et des soins anti-oppressifs pour favoriser un accès bienveillant et sûr à la contraception pour les jeunes. Ces résultats ont des conséquences pour les juridictions où les jeunes rencontrent des obstacles croissants à l’accès à la contraception.

## Resumen

Buscamos entender, ‘¿Cuáles son las experiencias, creencias, actitudes, conocimientos y necesidades de acceso a métodos anticonceptivos de las personas jóvenes de Canadá, desde las perspectivas de la juventud y los prestadores de servicios de salud?’ Exploramos servicios de anticoncepción centrados en la persona y destacamos la importancia de fomentar confianza, autonomía y consentimiento informado. Realizamos entrevistas semiestructuradas individuales con jóvenes (de 15 a 25 años) y prestadores de servicios de salud (PSS) por todo Canadá, que representan una diversidad de entornos rurales y urbanos, género, edad y etnias. Utilizando la teoría fundamentada constructivista social, identificamos temas clave relacionados con la autonomía reproductiva, la confianza y el consentimiento informado. Tras analizar datos de 79 jóvenes y 27 PSS, identificamos cinco temas: (1) Valorar la toma de decisiones compartida; (2) “Ejercer presión” a favour de la anticoncepción: poner fin al paternalismo; (3) Buscar atención antiopresiva; (4) Los servicios de anticoncepción se basan en la confianza; (5) Ejercer control sobre mi cuerpo. Los participantes valoraron la toma de decisiones compartida y querían sentir que “todos estamos juntos en esto”. Tanto los jóvenes como PSS participantes relataron historias de PSS que “ejercen presión sobre la juventud para que use anticoncepción” y experiencias de discriminación médica que impedían sentimientos de seguridad y autonomía. En respuesta, las personas jóvenes abogaron por atención antiopresiva, identificaron la importancia de “forjar relaciones terapéuticas” e hicieron hincapié en que fomentar la seguridad con PSS “debe basarse en la confianza”. Por último, la juventud relató practicar abogar por su autonomía reproductiva para tener control de su cuerpo. Nuestra investigación destaca la importancia de la toma de decisiones compartida, el consentimiento informado y la atención antiopresiva para fomentar acceso solidario y seguro a la anticoncepción para las personas jóvenes. Los resultados tienen implicaciones para jurisdicciones donde la juventud enfrenta cada vez más barreras al acceso a la anticoncepción.

## Introduction

*A*ccess to contraception is a human right^1^ that promotes bodily autonomy and upholds reproductive justice principles by allowing people to time and space their pregnancies, reduce the risk of sexually transmitted infections (STIs), complete their chosen education and career goals, build sexual freedom, and experience non-contraceptive benefits such as menstrual suppression and menstrual pain reduction.^2–5^ Persistent barriers exist for youth to access contraception in Canada,^5–7^ limiting their reproductive autonomy.

Reproductive autonomy refers to people’s right to make independent decisions about their reproductive health, including choices about contraception, free of coercion and violence.^1^ Conversely, reproductive coercion involves manipulation or pressure to control someone's reproductive decisions, including contraceptive coercion such as forcing, preventing, or sabotaging contraception use from sexual partners, family members, or healthcare providers (HCPs).^8,9^ Canada has a long history of reproductive coercion as a tool in the colonisation of Indigenous peoples, who have routinely experienced reproductive violence alongside Black people, people of colour, and disabled people, including sterilisation, forced abortion, and other interventions without consent.^10–12^ Beyond overt coercion, guidance or undue influence from individuals in the context of a power imbalance (e.g. patient + clinician; youth + caregiver) can undermine reproductive autonomy and independent contraceptive choice. For HCPs, taking a person-centred contraception care approach by being responsive to and respectful of clients’ needs, values, and preferences, protects against contraceptive coercion.^3,9^ This includes prioritising interpersonal connections with clients of care through trauma-informed relationship building as well as providing people with adequate information and using a shared decision-making approach.^13,14^

A 2023 survey of 1,154 reproductive-aged people in the United States (US) who were assigned female at birth (AFAB) found higher reported levels of psychological distress and worse mental well-being when people perceived coercion from their HCPs in their contraceptive care.^9^ A prospective study of clients from six San Francisco health clinics showed increased contraception counselling satisfaction when reporting a shared-decision-making model, with decreased satisfaction when they felt their provider had a contraception method preference.^14,15^ Other studies have found that different populations have varying experiences and preferences related to contraception counselling from HCPs.^13,16–18^ A recent systematic review by Manzer et al. summarised clients’ preferences for contraception care, including how person-centred approaches inform outcomes.^18^ Manzer et al. present results describing persistent disparities in who receives high-quality person-centred care, with people of colour, those with mental health and substance use concerns, disabilities, and lower incomes most impacted. Existing research is primarily from US settings and highlights the challenges of navigating systems of oppression and discrimination.

In a parallel systematic review examining HCP experiences providing contraception,^19^ including studies from Canada, the US, and 34 European countries, Manzer et al. also reported the important role of building strong interpersonal relationships with clients and focusing on specific client needs. The authors identified several barriers that hindered trust and person-centred care, including misconceptions about contraception methods and side effects, perceived lack of clients’ compliance, lack of formal contraception counselling protocols, limited time to provide comprehensive services, and language barriers which necessitated the use of translation services.^19^ Other studies have found that clinicians often adjust contraceptive counselling or method recommendations based on patient characteristics (e.g. age) or clinician preferences, inconsistent with a person-centred approach to contraceptive care.^20–23^ These results suggest the importance of relationship building, centring client’s preferences, and shared decision-making models as key aspects of high-quality contraception care.

The Canadian contraception landscape is rapidly shifting. Recently, British Columbia, Manitoba, the Yukon, and Prince Edward Island began offering free prescription contraception,^24,25^ and the Canadian government has passed legislation nationally to ensure free prescription contraceptives through Pharmacare.^25^ As of 2023, pharmacists are authorised to prescribe some or all contraception methods across all regions, excluding Manitoba, Ontario, and the Territories.^26^ Additionally, the COVID-19 pandemic catalysed increased telemedicine services, allowing for improved remote access to HCPs across North America.^27–29^ While free contraception reduces financial barriers, affordability does not equal access or acceptability. While the existing evidence in Canada suggests that youth face disproportionate barriers to accessing contraception, we lack an understanding of the lived experiences of youth seeking contraception in Canada, especially related to contraceptive coercion and shared decision-making.

In this context, it is critical to understand the contraceptive experiences and preferences of youth in Canada in order to support services that could optimise their reproductive health and well-being. In our study (Ask Us: Youth Voices to Improve Contraception Access),^30^ we seek to answer the question: “*What are the contraception access experiences, beliefs, attitudes, knowledge and needs of youth in Canada, from the perspectives of youth and youth service providers?*” In the present analysis we explore person-centred contraception care and highlight the importance of fostering trust, autonomy, and informed consent in models of care.

## Methods

### Study design

Our national qualitative study, the Ask Us Project, involved in-depth interviews with youth and healthcare professionals engaged in contraception care in Canada. The full study protocol^30^ and reflections on youth co-design of the study approach^31^ are published elsewhere. This project received ethical approval from the UBC Behavioural Research Ethics Board (H21-01091) on April 8, 2022.

### Critical frameworks

Our study was guided by integrated Knowledge Translation (iKT), a participatory research method that emphasises the involvement of knowledge users as equal partners throughout the research process.^32,33^ Our iKT approach aimed to enhance the relevance, appropriateness, and usefulness of co-generated knowledge; increase the potential for the research to be used by the public, HCPs, and policy makers in a timely manner; and ensure that young people exercised their power and expertise as equal decision-makers in all stages of the work. Thus, our team included researchers, youth living in Canada, HCPs, health professional associations (Society of Obstetricians and Gynaecologists of Canada, Canadian Paediatric Society), and government decision-makers. Youth research assistants (YRAs) led the day-to-day research activities: designing the interview guide, conducting participant recruitment, leading interviews, leading analysis, designing dissemination strategies, and co-authoring manuscripts.^31^

We employed a constructivist grounded theory approach to guide reflexive data collection and analysis.^34,35^ Constructivist grounded theory is a qualitative research methodology in which researchers use interviews and other qualitative data collection techniques to generate theories. We selected this method for its alignment with critical feminist, social justice-oriented, and participatory frameworks that attend to inequities and systems of oppression.^36^ To ensure that our interviews explored a range of “access” domains, our interview questions were informed by Levesque’s Patient-centred Access to Health Care framework^37^ and a health equity lens,^38^ and included probes for both the supply (approachability, availability, affordability, acceptability, and appropriateness) and demand (the ability to perceive, seek, reach, pay, and engage) domains of healthcare access (see Supplement 1). During analysis, we did not apply a conceptual framework and instead practised inductive coding for this exploratory study, to ensure results were grounded in participants’ lived experiences.

### Positionalities

The study lead (SM) is a straight cisgender woman, parent, and qualitative health services researcher who leads participatory research on contraception access. She mentored five YRAs (KJ, HS, AA, VP, RA) and a graduate trainee (ZK) to conduct the research activities. The YRAs are a group of mixed-race and white cisgender women and nonbinary youth, both queer and straight, who are first or second generation immigrant and refugee settlers in Canada and who identify as racialised individuals (people who experience the effects of racism because society categorises them according to perceived racial differences). Following a reflexive approach, we continually considered how structural inequities may shape the researcher-participant relationship and power dynamics. While some YRAs had prior experience working in research or social support positions with vulnerable populations, they had minimal or no prior experience in conducting research interviews. They participated in iterative group-based and one-to-one mentorship in the study methods and critical frameworks with SM for six months using a “capacity bridging” approach,^30^ which acknowledges that all team members bring expertise to the collaborative research and that the goal is to identify and “bridge” these diverse skills and knowledge. This led, for instance, to young people “bridging” their knowledge of trauma-informed care in community settings to design the trauma-informed research practices we applied in the Ask Us Project (see Supplement 2). All YRAs completed standardised ethics training and received continuous mentorship and support from experienced qualitative researchers SM and ME throughout the project.

### Setting and participants

We define “youth” as individuals in the developmental stage of emerging adulthood, when young people engage in identity exploration and development in order to transition into their personal and professional lives as adults.^39^ Participants included (a) young people aged 15–25 who have ever used, wanted, or considered contraception, and (b) HCPs who provided contraceptive care to youth. We included individuals of all genders who currently resided in Canada and were comfortable having a conversation by phone or videoconference in English or French, or in their preferred language with a translator present.

### Participant recruitment

YRAs (KJ, HS, AA, VP) and graduate trainee (ZK) designed all public-facing study materials^31^ and led a two-phase recruitment strategy involving (1) purposeful sampling to engage a diverse demographic of participants and (2) theoretical and snowball sampling to confirm/disconfirm results, fill in data gaps, and refine our evolving theory. Recruitment posters and social media posts were circulated in English, French, Spanish, Mandarin, Punjabi, and Arabic to enhance the reach and diversity of the sample.

For youth recruitment, YRAs engaged youth-serving organisations using principles of relational mapping with interest holders.^40–43^ We first conducted an environmental scan to identify youth-serving organisations focused on improving health and wellbeing for young people in equity-seeking groups. We prioritised organisations that served low-income, housing-insecure, rural, immigrant, racialised, and/or queer, trans, and gender-diverse youth.

We identified and partnered with 191 unique organisations across all provinces and territories, providing them with a social marketing campaign guide with instructions on how to disseminate the materials (see Supplement 3). The organisations then distributed paper and digital versions of the youth-designed recruitment materials. We maintained an ongoing connection with partnered organisations throughout the study and continued to explore mutual benefit opportunities through sharing project updates and resources including an infographic on Trauma Informed Practices (see Supplement 2).^44^ YRAs tracked outreach efforts and documented recruitment reflections. For HCP recruitment, we distributed the study information through email listservs of health professional organisations, youth sexual health clinics, sexual and reproductive health organisations, and online family planning communities of practice.

Recruitment began in October 2022 and participants were screened and interviewed on a rolling basis through December 2023. Interested participants reviewed and completed the online consent form and demographic survey hosted on the Research Electronic Data Capture platform (REDCap).^45^ We collected participants’ self-reported identity characteristics and contraception access/provision experiences, which informed our purposeful sampling technique. A research coordinator (AW) conducted videoconference screenings to confirm participants’ eligibility and then scheduled youth-led interviews over Zoom, phone, or in-person based on participant preference.

To support recruitment of youth aged 15–18, we onboarded a secondary school co-op student as an additional YRA (RA). She provided guidance on engaging teenagers in the study, co-conducted interviews with another experienced YRA team member, and contributed to data analysis.

### Data collection

After completing their training, YRAs conducted one-on-one semi-structured interviews with youth, individually or in pairs. The study lead (SM) interviewed healthcare professionals. Participants provided informed consent online prior to participation. We completed interviews over Zoom or phone. The interview guide included open-ended questions with follow up probes that considered equity and domains in the Patient Centred Access to Care framework,^37,38^ and the flow of conversation was determined by the participant’s input (see Supplement 4). After the completion of each interview, we wrote memos to document emerging patterns and relationships, which informed initial themes and the record-keeping of the analytical process. We did not have a predetermined sample size and continued to conduct interviews until we reached saturation through informational redundancy^46^ (i.e. when no new ideas are expressed in the interviews and the observed ideas cannot be used to create new themes or codes).

### Data analysis

The audio-recorded interviews were professionally transcribed verbatim by a third party. We assigned pseudonyms for each participant and indicated their age through the results as “XXyo” (years old). Data analysis was youth-led to ensure consistency with our iKT approach. We sought to make the analysis a tactile, participatory process, prioritising in-person coding workshops with paper-based transcripts and colourful concept mapping on poster paper to ensure rich discussion and reflections with the YRAs. Our analysis, guided by constructivist grounded theory, followed four steps: (1) open and *in vivo* coding; (2) focused coding into categories; (3) creating visual concept maps on paper and on a digital whiteboard to identify patterns and relationships between emerging categories; and (4) theoretical coding by categorising concepts into clusters.^34^

We engaged in multiple verification strategies to ensure the trustworthiness and credibility of the results of the analysis. First, we sent each participant a study update and an invitation to review their transcript for accuracy. Out of 79 youth participants, three reviewed their transcripts. Second, we conducted collaborative reflection with the YRAs through concept mapping workshops. This approach sought to ensure co-construction of analysis and empower YRAs to be active participants in shaping the final output.

## Results

We screened 100 individuals for the youth sample, of which 6 were lost to follow up and 11 were excluded during videoconference screening for misrepresenting their age or geographic location to meet study criteria to receive a study incentive. We interviewed 83 youth participants and excluded four from the analysis due to suspected fraud based on inconsistent responses and/or responses not matching the demographic profile they provided at screening. All HCPs who sought to participate met eligibility criteria and none were lost to follow up. In total, we included 79 youth and all 27 HCP interviews for analysis.

Youth participants were aged 15–25 years old. The majority identified as women (72%) and lived in urban areas (94%). HCP participants were primarily nurses (nurse practitioners, registered nurses, and public health nurses; 56%) or physicians (37%), identified as women (91%), and lived in urban areas (93%). Table 1 details the demographic characteristics of all participants.

**Table 1.** Characteristics of youth and HCP participants of the Ask Us Project

Characteristics	*n* (%)
Youth participants (*n* = 79)	
Age (yr)	
15-–17	7 (9)
18–20	19 (24)
21–23	30 (38)
24–25	23 (29)
Rural^a^	5 (6)
Urban^a^	73 (94)
Born in Canada	50 (63)
Born outside Canada	29 (37)
Sex assigned at birth	
Female	67 (85)
Intersex	0 (0)
Male	11 (14)
Prefer not to answer	1 (1)
Current gender identity	
Genderfluid	1 (1)
Genderqueer	2 (3)
Man	11 (14)
Nonbinary	6 (8)
Woman	57 (72)
Third gender	0 (0)
Two-Spirit	0 (0)
Unsure/ questioning/ undecided	2 (3)
Sexual orientation^b^	
Heterosexual	35 (44)
Homosexual	2 (3)
Bisexual	18 (23)
Pansexual	5 (6)
Queer	12 (15)
Asexual/ ace	1 (1)
Other	1 (1)
Don't know	3 (4)
Prefer not to answer	2 (3)
Current living situation	11 (13)
Living alone	33 (42)
Living with family	23 (29)
Living with roommates	11 (14)
Living with partner	1 (1)
A different living arrangement	0 (0)
Racial or cultural groups^c^	
Afro-Caribbean	2 (3)
Arab	3 (4)
Black- African	8 (10)
Chinese	11 (13)
European	12 (15)
Filipino	2 (3)
Indigenous	6 (8)
Japanese	1 (1)
Korean	0 (0)
Latin American	0 (0)
South Asian	12 (15)
Southeast Asian	4 (5)
West Asian	7 (9)
White	32 (41)
Other	4 (5)
Prefer not to answer	1 (1)
Highest level of completed school	
Grade 8 or lower	0 (0)
Grade 9 – 10	8 (10)
Grade 11 – 12	4 (5)
Secondary school	10 (12)
Some postsecondary studies	18 (23)
Trades certificate	1 (1)
Non-university certificate/diploma	6 (8)
Bachelor's degree	27 (34)
Graduate Degree	5 (6)
Insurance coverage^d^	
Yes	45 (57)
No	20 (25)
Don't know	11 (14)
Prefer not to answer	3 (4)
Contraceptive methods used^c^^,^^e^	
Copper IUD	2 (3)
Hormonal IUD	14 (18)
Contraceptive implant	7(9)
Birth control pill	54 (68)
Contraceptive patch	8 (10)
Contraceptive vaginal ring	3 (4)
Contraceptive injection	7 (9)
External condom	55 (70)
Internal condom	8 (10)
Withdrawal	34 (4)
Calendar method	65 (82)
Emergency contraception	13 (16)
Vasectomy/sterilization	24 (30)
Tubal ligation/hysterectomy	1 (1)
No method	1 (1)
Another method	9 (11)
Prefer not to answer	1 (1)
HCP Participants (*n* = 27)	
Profession	
Nursing^f^	15 (56)
Physician	10 (37)
Other^g^	2 (7)
Urban^a^	25 (93)
Rural^a^	2 (7)
Gender^h^	
Man	1 (5)
Non-binary	1 (5)
Woman	20 (91)
Age (Years)^i^	
<30	3 (12)
30–39	11 (42)
40–49	7 (27)
50+	5 (19)

^a^We defined urban participants as those located within Statistics Canada’s defined census metropolitan areas (CMA), with all other participants classified as rural. One youth participant did not provide postal code and urban/rural status was unable to be determined.

^b^Question constructs were co-designed with YRAs and included the following definitions: Heterosexual (I am attracted to people of the opposite gender as me), Homosexual (I am attracted to people of the same gender as me), Bisexual (I am attracted to people of the same gender and other genders as me), Pansexual (I am attracted to people regardless of their gender).

^c^Check all that apply questions.

^d^Coverage for all or part of the prescription medication.

^e^Methods them or current sexual partner have ever used.

^f^Includes nurse practitioners, registered nurses, and public health nurses.

^g^Other includes registered midwives and youth program facilitators.

^h^Confirmed via email after interview completion. Five participants did not respond.

^i^One participant did not provide their age.

Interviews ranged from 40 to 90 minutes and all were conducted over Zoom. Our results of analysis include five inductive core themes, summarised in Table 2: (1) *Valuing shared decision making*; (2) “*Pushing*” *contraception: putting a stop to paternalism*; (3) *Seeking anti-oppressive care*; (4) *Contraception care is all about trust*; (5) *Practising control over my own body.* Illustrative example quotes can be found in Supplement 5.

**Table 2.** Summary of key themes from qualitative interviews about youth experiences with contraception from the perspective of youth and HCPs providing youth contraception care

Theme	Sub-theme
Valuing shared decision-making	Youth want to focus on what matters most to them
	Making a decision with sexual partners
“Pushing” contraception: Putting a stop to paternalism	/
Seeking anti-oppressive care	Therapeutic relationship building
Contraception care is all about trust	Trusting family, friends, and community
Practising control over my own body	Learning to advocate for myself

### Valuing shared decision-making

Youth participants identified several attributes of shared decision-making, including rectifying imbalances in information exchange between youth and clinicians to focus on the factors that matter most to youth; making decisions about contraception methods in collaboration with their sexual partner(s); and making contraception choices with support or independence from family members.

#### Youth want to focus on what matters most to them

Youth and HCP participants shared that HCPs primarily focused youth contraception counselling on the effectiveness of methods for preventing pregnancy. HCPs frequently assume pregnancy prevention as young people’s reason for accessing contraception and overlook other factors that youth identified as equally or more important, including non-contraceptive indications (“I don’t want to menstruate” – Hanna, 22yo; “It helps them with their acne” – Bree, 24yo), side effects, ease of use (“I feel like I was very misled into thinking [IUD placement] would be a basic procedure” – Zahara, 24yo), and familiarity with the method.

Many HCPs, however, expressed clear interest in understanding what matters most to individual youth, for instance by trying “to let the patient guide the conversation” (Melissa, Physician). In counselling youth, several sought to “take the time to adopt a more holistic and educational health promotion approach” (Jasmine, Physician), recognising that youth are different from their adult counterparts in terms of experience and communication preferences. A nurse practitioner, Julia, described how being a salaried worker creates the conditions for her to take the necessary time to build relationships with youth and answer their contraception questions. This was especially important for contraception care because of the complexity and iterative nature of young people’s decision-making process, resulting in the need for longer or repeat appointments.

Other participants described how structural limitations like fee-for-service models hinder HCPs from prioritising relationship building and contraception counselling: “in a fee-for-service clinic, there would not be that time to actually devote to that education piece” (Sabrina, Physician).

Participants identified that HCPs providing step-by-step explanations are a key aspect of building safe relationships and encouraging informed consent for youth accessing contraception. As Salma (24yo) says: “[My doctor] even sat down and went through the entire little booklet that goes inside the pills, with me. She definitely helped me make a very informed decision.” HCPs described how they use drawings, diagrams, and anatomical models to visually explain contraception options as well as procedures like IUD placements so youth can better understand what is being put in their bodies.

Several participants emphasised that shared decision-making should not end at the initial appointment; it is equally important during follow-up to encourage youth to provide feedback about their contraception, and switch or discontinue a method as needed. As one HCP put it, “if you don’t like [the contraceptive method], you can call us back” (Lucille, Registered Nurse).

#### Making a decision with sexual partners

Young people’s experiences illustrated that *making a decision with sexual partners* was critical for contraceptive autonomy. Several youth explicitly framed contraceptive decision-making as a collaboration with their partners, where “it’s a decision we both make” (Joseph, 23yo) involving “open and honest communication” (Lee, 23yo). Others framed conversations about contraception with sexual partners as a way to build trust and respect: “because there’s a notion of respect between the two partners … there’s a notion of health, which is really reassuring” (Rowan, 19yo).

Other youth preferred to rely on methods they used independently of partners, in some instances because of a lack of trust. They felt more secure relying on methods that they could control. For participants who were assigned male at birth (AMAB), this often consisted of using condoms, regardless of their partner's contraception use, like Jacob (23yo): “I don’t know if they’ve been keeping up with their birth control intake or if they’ve been skipping days”. Many youth felt that taking responsibility for their own protection was the most effective way to ensure their safety, and regardless of gender or sex, they made sure to keep condoms on hand: “I just don’t always trust them, which is why I kind of just have [condoms] as backup” (Divya, 25yo).

### “Pushing” contraception: putting a stop to paternalism

Participants offered insight into the factors that led to “informed consent” for contraception, which they described as an ongoing and holistic practice that included discussions of method effectiveness, side effects, mental health implications, financial cost, placement and removal techniques (when applicable), and switching methods. While many HCPs shared concrete examples of comprehensive contraceptive counselling, many HCP participants recounted themselves or other HCPs “pushing contraception” (Lucille, Registered Nurse) or specific methods on youth. Another HCP reflected, “young moms who have given birth felt immense pressure, almost like an IUD was thrown into them after birth” (Fern, Public Health Nurse).

Youth participants also gave examples of feeling “pushed” to use, not use, or choose specific contraception methods during their healthcare appointments. One youth, Colette (23yo), explained that their doctor was unsure about prescribing an IUD, “so she pushed me towards the pill and that’s the only regret I have about when I decided to go on the pill because I really would have just preferred to go straight to the IUD, but it was like my doctor’s bias towards that.” Sage (25 yo) shared, “I had another physician of mine just keep constantly pushing birth control on me even though I wasn’t sexually active,” even after explicitly sharing that they “didn’t need it at this time”. In discussing youth contraception counselling approaches, many HCPs described walking the “fine line between enthusiasm and coercion” where method recommendations could be perceived as pressure (Sara, MD). HCPs, who perceived long-acting reversible contraceptives (LARC) to be the gold standard for preventing pregnancy, described downplaying their side effects for fear of youth being discouraged from using LARC methods. One HCP shared how “there are times when we adjust how we communicate with clients based on what we think is best for them, and I think we’re definitely at risk for doing that, often with the best of intentions” (Lena, Nurse Practitioner).

Some youth participants perceived that HCPs were “gatekeeping” (Hanna, 22yo) contraception methods. For instance, Bree, a 24yo Indigenous trans woman, described how youth face barriers accessing tubal ligation and vasectomy: “I didn’t want to be a parent, so I knew from a young age that I wanted a vasectomy. I had to wait till I was 18 until I had the legal authority over my own body to make that decision for myself, but I actually wasn’t even able to get a doctor on my side”. HCPs also observed examples of gatekeeping in their own practices and with peers, through placing conditions on access to or refills of contraceptive methods: “you used to only prescribe three months at a time or six months at a time … It was like this real … gatekeeper kind of attitude” (Heather, Nurse Practitioner).

In other cases, youth felt pressure from family members who oversaw their contraception choices. At a system level, many youth felt that their healthcare “was mostly under [their] parent’s control” (Isuri, 22yo), which highlights the challenges of navigating contraception choices as a dependent. Zoya (24yo) explained that their mom was “very hesitant about using things like birth control” and that their family “just had a lot of those cultural, religious restrictions, it made it really difficult to have open conversations with them because it would just be shut down almost immediately”. In more extreme examples, youth described how parents restricted their access to contraceptives or forced them to use contraception, sometimes specific methods. One registered nurse, Lucille, shared a story where a youth came for a Nexplanon insertion but showed signs of discomfort during the appointment. Lucille recalled that the patient revealed, “‘[I’m here] because my mom is worried I’m gonna get pregnant, and she is making me put this in my arm.’”

In another extreme case, Bree’s (24yo) father sabotaged their sister’s contraception by intentionally tampering with her condoms. Bree explained that “he popped a hole in them with an earring … it was very, like, a sneaky damage. Like, you wouldn’t know that this thing is now useless.” Bree went on to describe how this instance reinforced the “purity culture” in their upbringing that insisted that “abstinence is really the only true protection available.”

Youth also felt “pushed” by sexual partners who controlled or pressured their contraception use. Amy, a physician shared a disclosure from a youth patient: “I found out [my partner] has been sabotaging my birth control pills”, while a youth participant shared that the decision to go on oral contraception “was really my boyfriend pushing me to do it … He really pressured me to be on the pill” (Raina, 24yo).

HCPs shared their strategies for protecting against paternalism and contraceptive coercion, including asking screening questions and providing counselling about consent. A physician, Jasmine, described bringing up potential pressure to use contraception with youth, “I don’t always directly ask, but if the youth presents on their own and asks about birth control like, ‘Is there anybody that you’ve talked about this with?’ or ‘Is anybody making you feel pressured to go on birth control?’”

HCP accounts demonstrated that many changed their approach with young people over their lifespan. HCPs often offered more in-depth counselling for youth who were getting started with their contraceptive journeys. One nurse practitioner, Heather, described taking time in a step-by-step approach with a first pelvic exam, describing anatomy, and giving youth their speculum to hold and touch, to decrease fear. In contrast some HCPs described a perceived need to simplify information and “keep things tailored to a shorter attention span” (Lena, Nurse Practitioner) to keep younger youth engaged.

Other HCPs described approaching contraception counselling similarly across all age groups, stating that “if [youth] are old enough to have sex, they are old enough to talk to me about contraception” (Stephanie, Physician). Many HCPs adjusted their contraception counselling to meet the unique needs of youth who had children, those with disabilities, and those from cultural and racial minorities. Heather (NP) shared that “I am never going to pretend that I understand the nuances of all the different cultures, so I tend to be pretty frank with folks that are from visible minorities like, ‘Is there anything about this that would be difficult for you from a cultural perspective?’” Youth, in turn, recognised how HCPs approached contraceptive counselling in their own care, some with positive experiences of youth-oriented counselling, some with concern. One participant reflected on their first prescribing experiences where the HCP treated them “like a kid … Part of her prescribing me the birth control was her talking to me essentially through my mom instead of talking to me directly” (Isuri, 22yo). Others appreciated having providers meet them where they are at, as described by Megan whose HCP “explained it in a way that my grade 10 brain could understand it”.

### Seeking anti-oppressive care

Youth and HCPs highlighted a need for care grounded in anti-oppression principles that critically examines power imbalances to address health inequities, spanning individual, organisational, and broader policy contexts. They described what makes care anti-oppressive for young people, for instance in micro-level interpersonal situations by challenging power imbalances and attending to past or hidden traumas, and at a macro health system level by dismantling systems of oppression and correcting systemic and historical injustices. Youth stories identified a range of oppressive systems or “-isms” that impacted their care, including racism, sexism, transphobia, sexual violence, weight stigma, and ableism.

One key example centred on gender discrimination in contraception care. Trans and nonbinary youth commonly felt dysphoric in healthcare spaces and some had been misgendered while accessing care. Marty (25yo), a trans man, stated that “going to the gynaecologist is already quite stressful because of the femininity around it … so that’s quite dysphoria inducing for me”. Stephanie, a physician, highlighted the intersecting challenges of being in highly gendered spaces as well as navigating age hierarchies that trans and nonbinary youth can experience: “[I have] misgendered people in my clinic as well. Then afterwards, I realised and am like, ‘Oh, crap,’ but because they’re young, they didn’t correct me”.

Youth shared stories of trying to access contraception for menstrual suppression, both for gender affirmation and menstrual symptoms. HCPs often tried to redirect the conversation to pregnancy prevention or did not know how to appropriately provide care for menstrual suppression. Hanna, 22yo, who was questioning their gender at the time of the interview and was not sexually active with a person who can produce sperm, shared “every time when I would try to access my refill or just picking it up, I would have questions be like, ‘Do you need a pregnancy test?’ … I was a little bit dysphoric at that point. It came to me that people are still seeing it as just a contraception option and not so much menstrual suppression.”

Some youth described experiences of medical mistreatment including HCPs being dismissive, condescending, and discriminatory to youth clients. Participants perceived that actual or anticipated side effects to contraception are “disregarded by medical professionals” (Casper, 22yo). Divya (25yo) described how “women’s pain tends to get dismissed or undertreated” highlighting, for example, the need for people to know “whether or not they’re going to get numbing” before IUD placements. Another, Charlie (25yo), felt like they were being treated as a “hysterical teenage girl, which I’m currently none of those things” when advocating for their needs. One key example of power imbalances and systemic injustice was in youth being denied access to permanent contraception due to a perceived lack of maturity. Bree (24yo) described how their sister was withheld tubal ligation “because she wasn’t married”.

Across participants, there was a call for increased consideration of mental health in contraception counselling, including the positive and/or negative mental health impacts of using contraception. Youth desire that HCPs “acknowledge[e] that sexual rights and reproductive health are also closely linked to all of the other domains of health and they just don’t live in isolation, especially for mental health” (Lianne, 22yo). Additionally, youth shared that contraception counselling in itself impacts their mental health. Arnie (22yo) described an experience seeking emergency contraception where a pharmacist commented on their weight when choosing which pill to prescribe, stating that “it really felt like there was no privacy … Had they brought me to a private little consultation room or something, I feel like that might have eased my anxiety”.

Finally, experiences illustrated a systems-level need to address racism and colonisation in the healthcare system: “I felt like I was a second-class citizen because the colour of my skin” (Malina, 24yo). Sara, an Indigenous MD, spoke about the histories of “forced sterilisation of Indigenous women” and the presence of anti-Indigenous racism in contraceptive care, highlighting that “it’s very important to know that context when working with Indigenous people”.

#### Therapeutic relationship building

HCP participants offered examples of anti-oppressive care that were characterised primarily by *therapeutic relationship building*, or taking the time to build trust, rapport, and safety for youth. This included asking questions about young people’s lives, explicitly asking about safety in relationships, and engaging in shared decision-making practices. For instance, Marin (Public Health Nurse) described building rapport with youth patients by starting the conversation with their right to confidential care and consensual relationships. Similarly, Bella, a youth worker, emphasised the importance of ongoing relationships with continuity of care, supporting youth to know that they can safely come back to the care team at any time: “You hope that within the darkest moments, you’ll be the person that they reach out to. Just relationship-building connectedness and stuff, just reducing vulnerabilities, and increasing protective factors is really what we do”.

### Contraception care is all about trust

Participants highlighted that trust is essential between youth and the people they go to for reproductive care and information, including HCPs, family, partners, community members, friends, and teachers. As one youth emphasised, “it’s all about trust” (Cai, 18yo).

Some youth felt uncomfortable talking about sex and contraception with a familiar HCP and appreciated the anonymity of a new provider. For them, the trust was based on privacy. However most youth in our study felt that trust developed by going to a consistent person who had proven to be reputable and/or “knows all of my history” (Megan, 20yo). Specifically, for youth that did not have a primary care provider, it was challenging to find trustworthy contraception prescribers. They navigated around crisis pregnancy centres that blocked their access to contraception (Bree; 24yo) and walk-in clinics where they felt rushed, uninformed, and saw a different doctor each visit (Raina, 24yo). Additionally, youth often found it easier to build trust with HCPs that shared similar values or experiences (e.g. same gender, younger providers, shared identities, etc.). An (22yo), a newcomer youth from China, described that out of the ten or more HCPs they had seen in Canada, only two were Asian or Black, Indigenous, or people of colour: “I feel it’s the comfort of being your own skin and knowing that, ‘Oh, actually, I can relate to this person’ … it’s important to feel that you have an ally, and you have somebody that understands”.

HCPs and youth shared examples of how HCPs’ trust in patients impacts care. Some HCPs expressed their confidence in youth to make informed decisions around contraception: “they should be able to choose for themselves” (Marin, Public Health Nurse). Amy, a physician who is starting to be known as a provider “willing to provide permanent contraception to youth” is insistent that young people be respected in their choices. However, other HCPs were more hesitant to trust youth in their contraceptive choices: “I think, as a HCP, especially with young humans, we assume very easily that they don’t know yet what they want, so I think that’s a fallacy” (Lena, Nurse Practitioner). Lena continued to describe how “it came down to [her] ethics a little bit” when providing contraception care to clients who she perceived to be “partying and having unprotected sex” irresponsibly: “I felt really compelled to do whatever I could to help prevent babies from being exposed to alcohol in utero so that that cycle could be broken. It felt a little bit like hearing young women share their stories about partying and having unprotected sex, felt a little bit like watching child abuse … I felt like by offering them contraception, that was one small, if they wanted it, that would be one small part in trying to prevent the next generation being affected”.

A registered midwife, Nancy, described concerns around method choice and continuation, and how many youth clients would “just not come for their appointments … You write a prescription for them for a patch or OCPs or whatever, and they just never pick them up … which is a shame”. Nancy described an instance where a youth “had an IUD, and she pulled it out herself. She said it fell out in the shower, but none of us really believed her”. While some HCPs reflected on how social determinants of health could influence youth to avoid care or discontinue a method, few reflected on the role of youth mistrust in the healthcare system.

#### Trusting family, friends, and community

The concept of trust also extended to relationships with family, friends, and community as sources for contraceptive information. Megan (20yo) described having a complex medical history and sought comfort in their mom being part of her decision-making process: “[She] has always been very open to talk to me about all this type of stuff*”*. Brittany (22yo) shared, “I know I really trust [my sister], and I know that when she’s finding the information for me, she’s looking at the best resource”.

Conversely, many young people shared that they tried to hide their use of contraception from family and community members. Youth referenced how stigma, often cultural, religious, or generational, impacted how their families and communities approached conversations about contraception. This stigma often led to fear of punishment if they were caught using contraception or having sex and led them to creatively approach their own contraceptive care. For instance, a strategy many used was to pay for contraception out of pocket rather than access subsidised prescription contraception through their parents’ insurance plan in fear of their family finding out: “I would never in a million years use my parents’ insurance to cover it” (Leena, 25yo). Nadia (23yo) explained, “I am so scared of anyone in my life, any family member in my life ever finding out because it would actually be an explosion”. Nadia further described how they put their phone on airplane mode when having sex from fear of people finding out they are sexually active.

Pharmacies were also mentioned by youth when discussing trusted environments. Some frequented pharmacies farther away from home, either to avoid encountering people they know or to access a store with a self-checkout: “I would still drive to a store that has a self-checkout over going to a store that’s closer, but then I have to go through someone” (Bree, 24yo). However their trust in pharmacies being a confidential space was frequently compromised through poor “counter” experiences, for instance lacking privacy during assessments at the till (Arnie, 22yo) or anticipating “stigma” when requesting Plan B (Zoya, 24yo).

### Practising control over my own body

Youth desired reproductive autonomy and reported practising control over their own bodies through their contraceptive choices: “I feel like it’s my body, and I wanna make sure nothing happens” (Aisha, 19yo). For some youth, using contraception helped them feel in control, allowed them more reproductive freedom, and increased their sexual satisfaction. Whitney (22yo) described how condoms help them “completely eliminate a lot of anxieties I feel about sexual contact, mainly in the realm of like contracting or transmitting infection,” stating that “condoms are fucking awesome”.

Youth across genders desired more acceptable contraception options for people who were assigned male at birth, both so that they could have more contraceptive choice, and so that people who are assigned female at birth (AFAB) could more confidently choose to use or not use contraception as the responsibility of contraception would be more equitably shared. Zahara (24yo) shared, “It’d be nice to have male birth control since males could get several women pregnant at once”. Harry (24yo) expressed a strong desire to have more control in preventing pregnancy: “bring male birth control to the market, please … when you’re in control of it, it’s different”.

#### Learning to advocate for myself

Gaining reproductive autonomy also involved youth learning to advocate for themselves in healthcare settings. Youth described needing to learn self-advocacy skills with HCPs, family, and partners in order to feel heard. For instance, Lianne (22yo) described how they have learned to be more direct with HCPs, “in terms of like, ‘I know my body best, and my bodily autonomy should be respected. As a family doctor, you should be providing guidance and not directions’”. Similarly, Isuri (22yo) spoke about a conversation they had with their mother seeking to persuade them to recognise their contraception needs: “I was so young, I definitely didn’t understand what I was necessarily advocating for. It was me mostly just … being like, ‘Hey, it’s just for health purposes. It’s not anything sexual. This is a medication. It’s not necessarily for contraception’ even though it could have definitely turned into that”.

Several HCPs, in turn, supported youth in developing self-advocacy skills. Bella, a youth worker, described how they supported youth to speak up for themselves in the healthcare system: “Building self-esteem is a huge one. Educating them on their rights and advocating on behalf of them as well as teaching them how to advocate for themselves”. Lena, a community health nurse practitioner, shared an instance where a youth client took control and self-advocated to keep the conversation away from contraception. The youth had stated, “‘I wanna come and see you, but I don’t wanna talk about contraception. If you talk about contraception, if you ask me, I’m gonna walk out.’” In reflecting on that experience afterwards, Lena questioned, “you have to feel pretty coerced in order to have that reaction, right?”

## Discussion

In this qualitative exploration of youth experiences seeking contraception in Canada, we identified that youth desire reproductive autonomy and frequently experience barriers to accessing care, pressure to use or not use certain methods, and mistrust of HCPs and the health system. To address this, youth require anti-oppressive care that is rooted in building mutual trust with HCPs, family, and partners. Through trusted therapeutic relationships, youth reported feeling increasingly autonomous, confident, and informed in their contraception choices.

Our results indicate that there is opportunity for improvement in contraceptive counselling and care provision in Canada. HCPs need to consider youth as capable individuals whose perspectives, values, and rights to make decisions about their own bodies deserve respect. This aligns with the 2018 Canadian Paediatric Society position statement that was reaffirmed in 2024 which encourages HCPs to collaborate with youth to find a contraception method that feels “acceptable, safe, effective, and practical”.^7^ Despite many HCPs in our sample sharing this position, youth in our study described feeling “pushed” by HCPs to use contraception that did not align with their preferences.

Our results suggest that there is a disconnect in how contraceptive pressure or coercion is understood by young people and HCPs. Definitions of contraceptive coercion vary and are widely debated across the literature.^47^ This ambiguity could lead to instances where HCPs may provide well-intentioned recommendations based on what they believe is best for a client, while youth clients may feel pushed by HCPs to use a method outside their preference.^20–23,48^ A survey of 1,154 reproductive-aged AFAB people in the US showed that perceived contraceptive coercion from HCPs results in poorer mental health outcomes and an increase in distrust in HCPs.^9^ Other research demonstrates clinician bias in approaches to contraception care,^20–23^ including making normative assumptions when counselling sexual and gender minority youth on their contraception options.^49^ In line with our results, this highlights the importance of centring youth autonomy and consent in contraception decision-making.

Several AFAB participants described feeling like their contraceptive choices were compromised or controlled by partners or family. A survey of 370 racially and ethnically diverse female-identified participants aged 19–22 in Texas which explored experiences of dating violence found that 11.4% of study participants had experienced a form of reproductive coercion in a romantic or sexual relationship. When compared to other forms of intimate partner violence (IPV) experienced in the last year, participants reported 16.5% sexual IPV, 17.3% physical IPV, and 74.8% psychological IPV. Of the 42 participants who reported reproductive coercion, 34 reported experiencing another type of IPV.^50^ In our study, several youth and HCP participants described instances of IPV and reproductive coercion among youth, as well as contraceptive coercion from families. Youth shared instances ranging from parents intercepting youth from accessing contraception to parents and partners tampering with condoms and contraceptive pills, which may also be considered abuse or violence. Our results indicate the need for further research with young people to understand the range of pressures they may experience in their contraception decisions and potential interventions that can support reproductive autonomy.

We further identified that youth experiences of discrimination, including but not limited to transphobia, queerphobia, racism, and weight stigma, contributed to participants’ distrust in contraception health services. Similar experiences across broader age ranges are well documented in Canada and internationally. Recent studies exploring comfort levels in Canadian primary care highlight that queer and trans people report higher rates of discomfort than straight and cisgender people.^51,52^ Several studies across Canada and the US show that Black, Indigenous, and people of colour are more likely to experience contraceptive coercion and often already experience distrust with the healthcare system due to histories of medical racism and oppression.^10,53^ A study involving 24 women who identified as “overweight” or “obese” in Canada demonstrates how weight stigma negatively impacts fat people seeking reproductive care who are culturally “hyper-racialized”, “de-classed”, and “queered”.^54^ Our results illuminate how discrimination can impede young people’s confidence and willingness to engage with HCPs in fear of covert or overt biases and discrimination. Future population-level or survey studies may explore and test these findings in a larger sample of Canadians, to quantify how stigma and negative experiences influence youth access to their preferred contraception.

It is critical to consider these youth-specific findings as health systems improve access to safe and equitable contraception care nationwide. Participants described the perceived and experienced benefits of shared decision-making and identified a need for improved education for HCPs on topics including gender diversity, sexuality, race and racism, newcomer experiences, as well as skills to encourage consent and prevent coercion from family, partners, and themselves as clinicians. Promoting shared decision-making alone is insufficient to address these gaps, and comprehensive training for person-centred contraceptive counselling is required,^14^ as well as dismantling the provider bias and structural inequality that hinder true shared decision-making for contraception.^55^ In Canada, for instance, a synthesis of recommendations responding to recent cases of forced or coerced sterilisation of Indigenous women in Canada included anti-racism training recommendations at the provider and system level.^56^ For providers, training should include education on impacts of colonialism, systemic racism, harmful stereotypes, and their impacts on patient-provider contraception interactions. In parallel, health systems should increase the number of Indigenous healthcare providers and provide sexual health and healthy relationships education for Indigenous women in all Indigenous languages to build awareness and self-advocacy.^56^ These cultural safety recommendations echo the needs identified in our study, for anti-oppressive, person-centred contraception care with increased youth involvement in contraceptive counselling.

### Strengths and limitations

We meaningfully engaged with youth through an iKT approach including a co-design process to develop recruitment strategies, outreach to local community organisations, data collection, and data analysis. We employed relational mapping^30^ to develop ongoing collaborations with youth-serving community organisations across the country. This enabled us to recruit youth through a trusted environment as well as build partnerships with local organisations.

Participants were encouraged to complete the interview in a place and format that met their needs, including remote (videoconference and phone) and in-person interviews, with the option for a support person to accompany the interview at the participant’s request. All participants chose a remote interview option. Virtual data collection enabled us to conduct interviews nationally and allowed individuals to participate without incurring travel or transportation costs. Although all participants completed interviews in English or French, our recruitment materials were circulated in multiple languages with the option for participants to conduct the interview in their preferred language with a translator present.

Youth and HCP participants were recruited separately, and we are unaware of any HCP participants that provided direct care to the youth participants. This means that the contraception experiences that youth described may have little or no commonality with the care the HCP participants provide.

While many youth and HCPs discussed topics related to reproductive autonomy and discrimination such as racism, transphobia, or homophobia, contraceptive coercion, and medical discrimination, we did not introduce these academic terms or jargon in the space of the interview. We did, however, add an open-ended question related to discrimination after identifying this as a pattern in early interview responses*.* We also probed about decision-making, the role of culture and family, and the role of stigma in contraception access*.* This means that we may not have captured the full range of experiences of reproductive autonomy or discrimination of youth participants.

## Conclusions

Our results identify characteristics of person-centred contraception care and highlight the importance of fostering trust, autonomy, and informed consent in models of care. Creating resources, trainings, or toolkits that support therapeutic relationship building and decision-making between youth and HCPs could promote reproductive autonomy for youth in Canada. Further qualitative investigation of the perspectives and experiences of youth and HCP dyads could provide deeper insight into the similarities and differences in how reproductive autonomy and coercion are conceptualised. Our research also indicates the need to further examine the impacts of medical discrimination and the broader context of oppressive systems, including racism, patriarchy, misogyny, and cis-heteronormativity, to better capture and understand the experiences of equity-seeking youth.

## Supplementary Material

Supplement 5. Themes and representative quotes

Supplement 3. Social Media Guide 1

Supplement 4. Interview Guides 3

Supplement 1. Conceptual Framework

Supplement 4. Interview Guides 4

Supplement 4. Interview Guides 1

Supplement 2. Trauma Informed Guide

Supplement 4. Interview Guides 2

Supplement 3. Social Media Guide 2
