# Supplementary material for: Trust, autonomy, and informed consent: a qualitative investigation of youth experiences accessing contraception in Canada
Source: Sex Reprod Health Matters. 2026 Jun 1;33(1):2678063. doi: 10.1080/26410397.2026.2678063 (PMC13348105; doi:10.1080/26410397.2026.2678063)
Supplement: Supplement 5. Themes and representative quotes [file ZRHM_A_2678063_SM8667.docx]

## **Supplement 5**: Illustrative example quotes of key themes from qualitative interviews about youth experiences with contraception from the perspective of youth and healthcare providers providing youth contraception care.

| Theme | Sub-theme | Example Quotes |
| --- | --- | --- |
| Valuing shared decision-making | Youth want to focus on what matters most to them | “I’m gonna be honest. I think everyone goes through stuff, and all us patients want from a health team member is for someone to understand us and what our bodies are going through and be able to help us through that process. That’s honestly what I think about because I haven’t had a lot of help during my life. I’ve just kind of been on my own fending for myself, so it’d just be nice for once to have someone to help me through it because I never got, like, I never got told what a period was. I didn’t know what it’d do to you, what would happen, so I learned it all by myself. I just feel like I deserve at least some help and some push.” - Michelle (youth)  “I’m the luckiest person because, for me, my day is kind of about what patients need, not necessarily what a schedule looks like. I’m able to go into a room, spend an unlimited amount of time with somebody, answer every single one of their questions, and I’m not penalized for it.” – Julia (health care provider) |
|  | Making a decision with sexual partners | “I rely pretty heavily on open and honest communication as a workaround when certain forms of contraception aren’t super desired by either party ... I remember one time, I had a first date with a guy. We went to a bar. Then we were going to hook up, and we realized neither of us had a condom, so we didn’t have penetrative sex. We just grinded on each other and made out, and we had a great time. Lovely night. I think that’s something else that’s lacking from sexual education is just the creative bit around it. Think outside the box. There are many ways to enjoy pleasure and enjoy intimacy with someone else.” - Lee (youth)  “My partner who I’ve been to several appointments with has an IUD, things like that ... My partner has an IUD, so I kind of count that because I was there when it was put it, and I was part of that conversation, but not in my body.” - Charlie (youth) |
| “Pushing” contraception: Putting a stop to paternalism | / | “Coercion is big, right, not only in abortion care but contraception. When I first started eight years ago in abortion care, providers that I’ve worked alongside, some are pushing contraception, and it made the patient very, it made the patient uneasy and not comfortable and felt pressured and almost judged for being there because it was their fault that they got pregnant in the first place. That’s why I feel that it’s important to mention that, because I don’t want them to be pressured by any method. I want them to be aware and understand the side effects and how everything works and what to expect of each method so that there’s no surprises once they’ve started it. It’s (inaudible) or ‘Oh, she did tell me that, and it is normal. Can I live with it or not?’ or ‘She told me to wait it out for a couple of months to see if my body adjusted to it.’ I like them to be aware and that they’re in control.” - Lucille (health care provider)  “Sometimes I felt a little bit conflicted about someone who was sort of on the fence about using contraception, hormonal contraception … I would say, ‘There are some side effects. These are really important side effects to know about,’ but I would not go into all the details about the extent of nausea, yes, irregular bleeding is normal, but I wouldn’t embellish the side effects. I felt like I kind of tried to tailor my conversation with them. Sometimes it backfired on me, especially with Depo, when people were really upset that they had irregular bleeding even though we talked about it beforehand. If they said that they wanted Depo, I would give them Depo, but then sometimes they came back saying, ‘I will never go on that stuff again.’” - Lena (health care provider)  “I usually do take some time to just demystify the whole thing and draw what is the uterus, what is the cervix, what is the vagina, how is it different from the cervix kind of thing. That can help a lot ... I usually always ask if they’ve had a pelvic exam with a speculum before. If they haven’t, then I take it out of the package. We use metal speculums. I take it out of the package, and I show them. I have them hold it and touch it and, ‘This is not sharp. There’s nothing sharp or scary about this, but listen to this. It does sound quite scary, like clunk, clunk, clunk,’ and just trying to, like, because I think the whole procedure goes a lot smoother if the fear has been decreased.” – Heather (health care provider) |
| Seeking anti-oppressive care | / | “I’m a very type-A, research-oriented person, so I already knew quite a bit about the different birth control options that were available to me, but we talked them over. I’m also a plus-size woman, so unfortunately, my doctor, I was more interested in IUD, and unfortunately, my doctor would not recommend that for me because I am plus size. She has, I think, some pretty backwards notions about women’s health and getting on an IUD because it’s sometimes associated with weight gain, so I stayed on the pill just because I’ve had a really easy time with it.” - Lola (youth)  “I think the current Canadian healthcare system is very misogynistic, patriarchal, racist, sexist, and all of the systems have oppressions, so I think just changing the framework around it and integrating more Indigenous wellness and Indigenous methods of healing and coming from a more holistic lens with health and wellness is really gonna transform population health but also individual health.” - Aayana (youth)  “I grew up in foster care where it is very, hmm. I’m an Indigenous person, but foster care in [province] is predominantly White Christian as well as a very conservative environment, so positive sex talks aren’t commonplace, and access to contraceptives aren’t really allowed. It’s taboo, so something you could get in trouble for.” - Bree (youth) |
|  | Therapeutic relationship building | “We usually give the parameters of confidentiality and privacy in youth clinic. We get a full medical history and then just try to build that rapport, letting them [know] it’s a safe space. We want to build that relationship with [youth] so that they do continue to come back as well as touching on any of the like, ‘So, what are you up to these days? Are you at school? Are you working? Where are you living? How is that going? Oh, are you sexually active? Okay. Are you thinking about becoming sexually active? How is that relationship? Do you feel safe in it? Is this something that you’re consenting to?’ Just that whole kind of additional piece of healthy relationships as well.” -Marin (health care provider)  “If a young person, say, under 16 is coming—and that’s just an arbitrary number—but has made an appointment on their own, comes in, and says, ‘I’m having sex, and I think I should be on birth control,’ usually, one of my first statements would be around confidentiality like, ‘I’m so glad that you’re here. You’ve come to the right place. I can help you. Just so you know, the conversation that we have today and any actions that we decide to take is between you and I, but I do need you to know that if you are at any risk of harm, I’m gonna be required to involve a parent or a guardian. That being said, let’s talk about what you want to do. My job is to make sure that you’re making a decision that’s best for you’ kind of thing.” – Heather (health care provider) |
| Contraception care is all about trust | / | “I will typically see some South Asian, South East Asian patients who come for contraception care only or maybe they’ve had a medical abortion only and then typically have a family doctor but actually try to keep that separate because other family members go to the family doctor and are afraid of disclosure by the family doctor because there’s a lot of stigma, and there could be some negative consequences in the family structure if some family members find out that they’re seeing contraception, abortion care or are sexually active.” – Sara (health care provider)  “I would go to mainly my gynaecologist just because he knows all of my history with my cysts and stuff, so he knows how to protect my ovary very well, but I would say that any healthcare professional is quite trustworthy. I would still talk to a pharmacist or a nurse or a nurse practitioner or walk-in clinic doctor or my family doctor about it, but I would solidify things with my gynaecologist before switching or making any moves with my contraception.” - Megan (youth) |
|  | Trusting family, friends, and community | “I was also prescribed it with my mom in the room with me, so it took kind of awhile to get her to be convinced. Because of that, I think I brushed over wanting to know about the side effects necessarily because it was mostly just trying to convince her that it wasn’t for sexual purposes; it was solely for acne control. I think because of that, I didn’t take the health concerns quite as seriously as I should have. I eventually kind of stopped taking it because it had such a negative effect on my mental health.” - Isuri (youth)  “Yeah, so what I really like about it is that I know I really trust [my sister], and I know that when she’s finding the information for me, she’s looking at the best resources. She’ll spend hours going through research papers and just figuring out what the best thing would be for me. I know that she has my best interests at heart, yeah. I just like knowing that it’s tailored to me, and she knows all my medical history and all, obviously, my sexual activity and stuff, so it just feels really nice to have.” - Brittany (youth) |
| Practicing control over my own body | / | “What I like about [condoms] is that, for people who have a penis, it is one of the very few forms of contraceptives that we would have control over. Whereas a lot of contraceptive methods, again, the responsibility is placed with the person who has a vagina and/or uterus.” - Bree (youth) |
|  | Learning to advocate for myself | “I had to be so meticulous about the way that I was advocating for myself in order to have my mom get on my side. I think there was definitely advocating that was happening that might not have necessarily been true.”  - Isuri (youth)  “I think it’s hard when, like, I recognize the power dynamic between a medical professional and a patient. I think when you take on a patient in a sick role, you really feel the power imbalance with doctor or whoever the medical professional is. I think, as a 19 year old, like from a teenager perspective, I was just, frankly, just taking instructions from a medical professional, so I didn’t even have the thought to challenge a microaggression or proceed with a complaint or anything or address the microaggression immediately. I think it’s ironic because, at the time, I was on [a national youth advisory board], and one of the projects we worked on was helping youths understand our rights, the sexual rights and reproductive health-related medical procedures.’” - Lianne (youth) |
