# Supplementary figures and images for: Trust, autonomy, and informed consent: a qualitative investigation of youth experiences accessing contraception in Canada
Source: Sex Reprod Health Matters. 2026 Jun 1;33(1):2678063. doi: 10.1080/26410397.2026.2678063 (PMC13348105; doi:10.1080/26410397.2026.2678063)

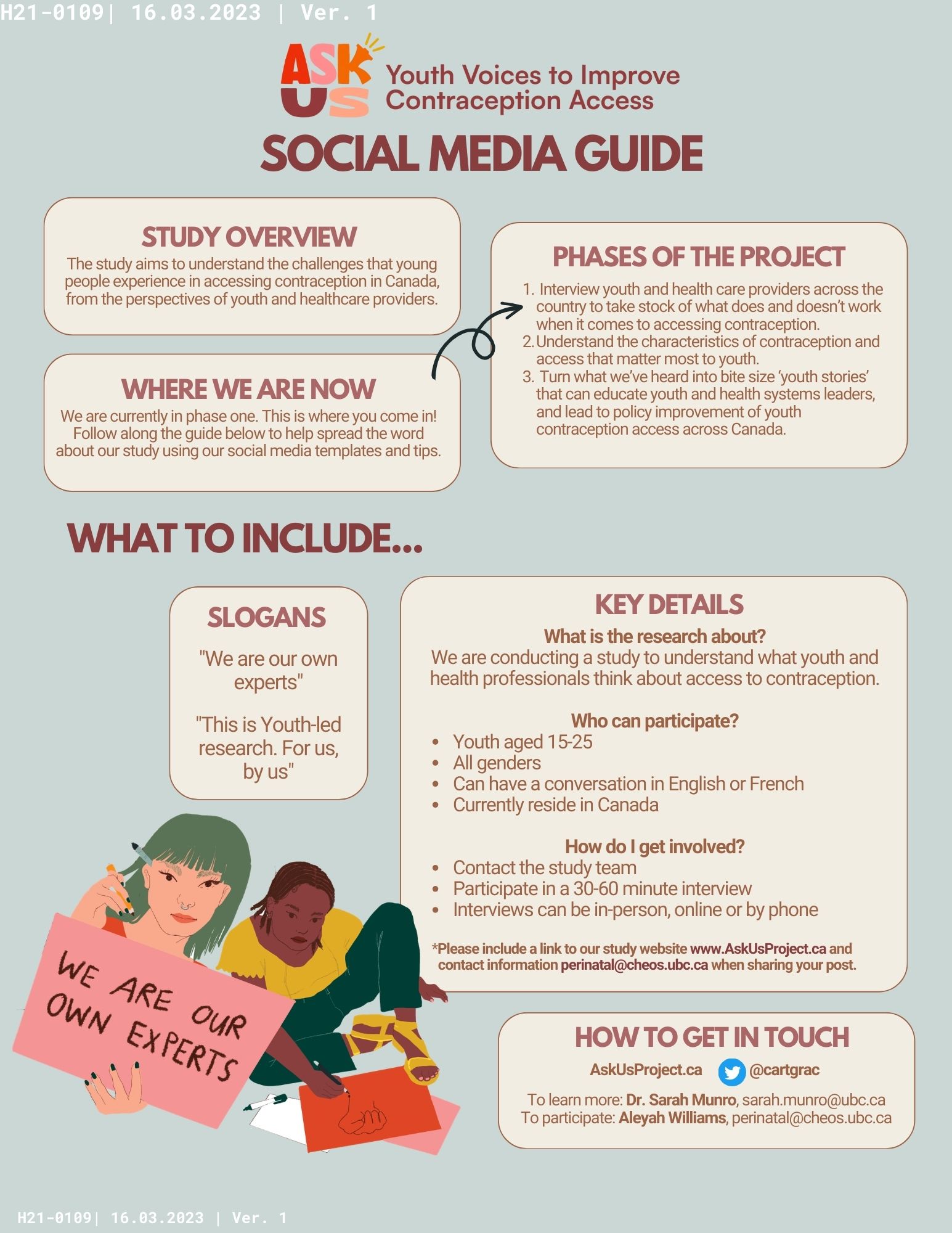

Supplement: Supplement 3. Social Media Guide 1 [file ZRHM_A_2678063_SM8666.jpg]

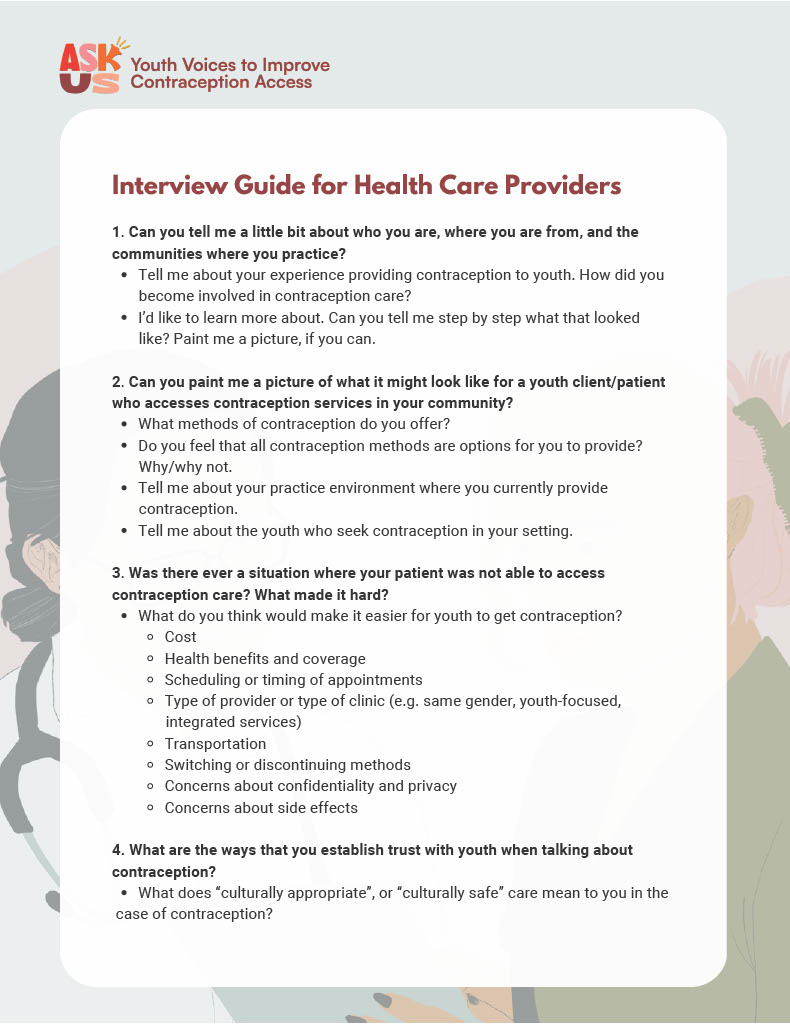

Supplement: Supplement 4. Interview Guides 3 [file ZRHM_A_2678063_SM8665.jpg]

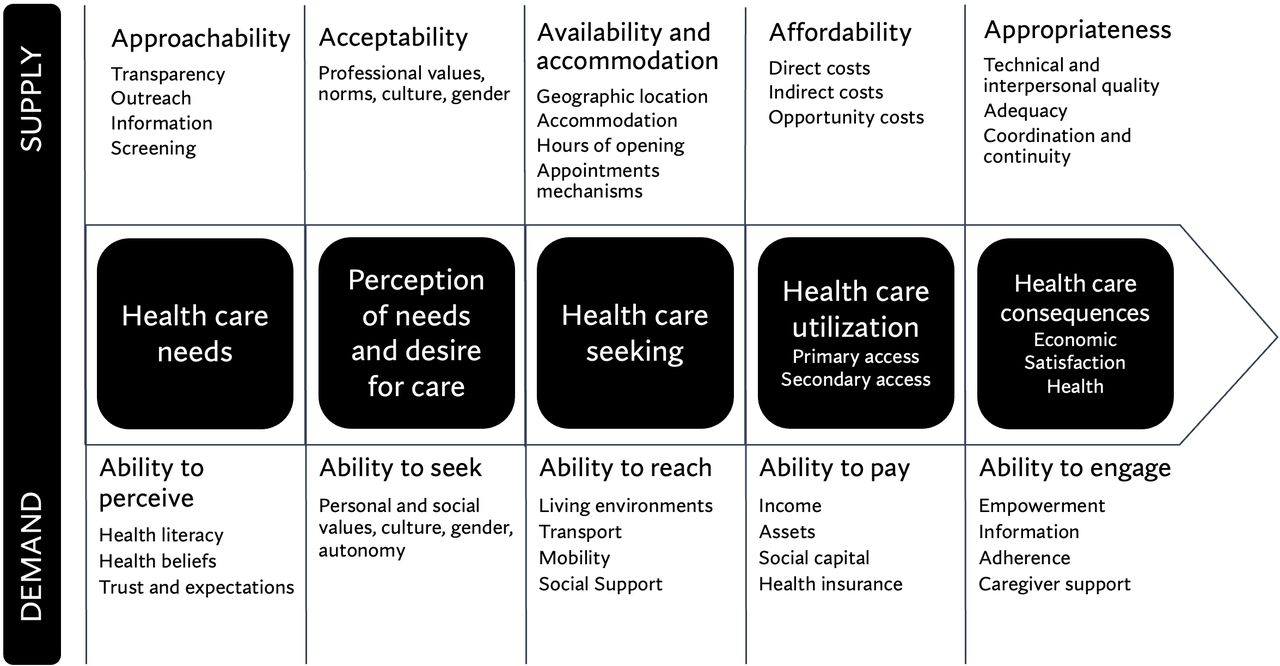

Supplement: Supplement 1. Conceptual Framework [file ZRHM_A_2678063_SM8664.jpg]

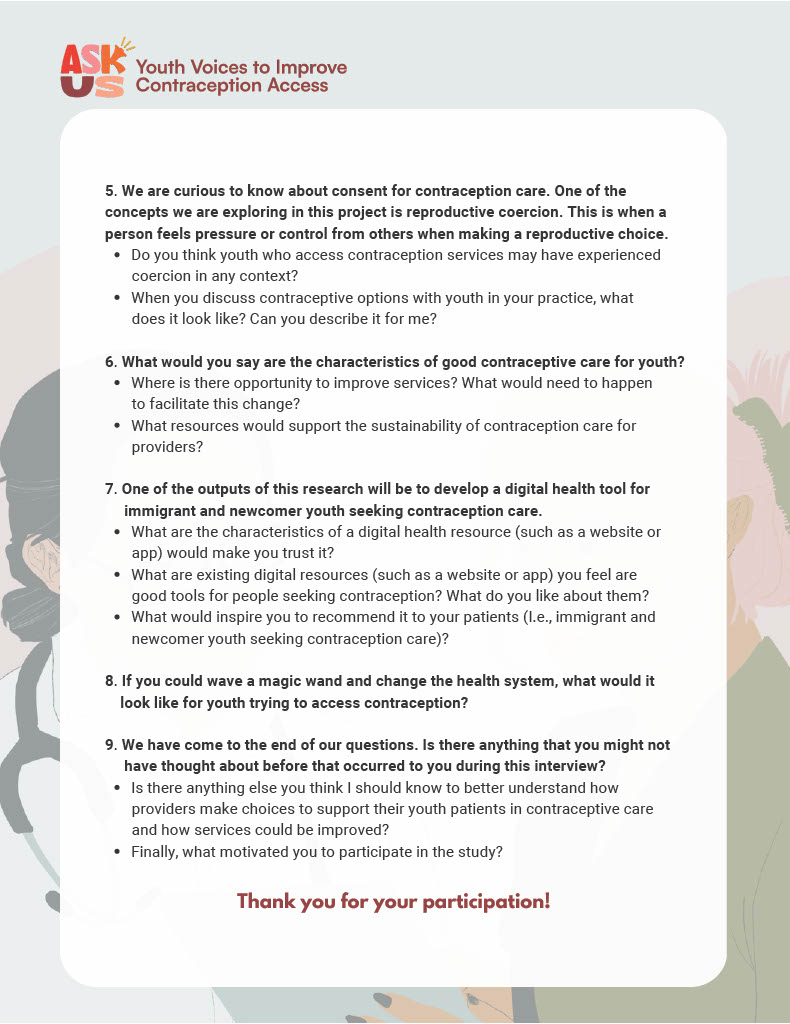

Supplement: Supplement 4. Interview Guides 4 [file ZRHM_A_2678063_SM8663.jpg]

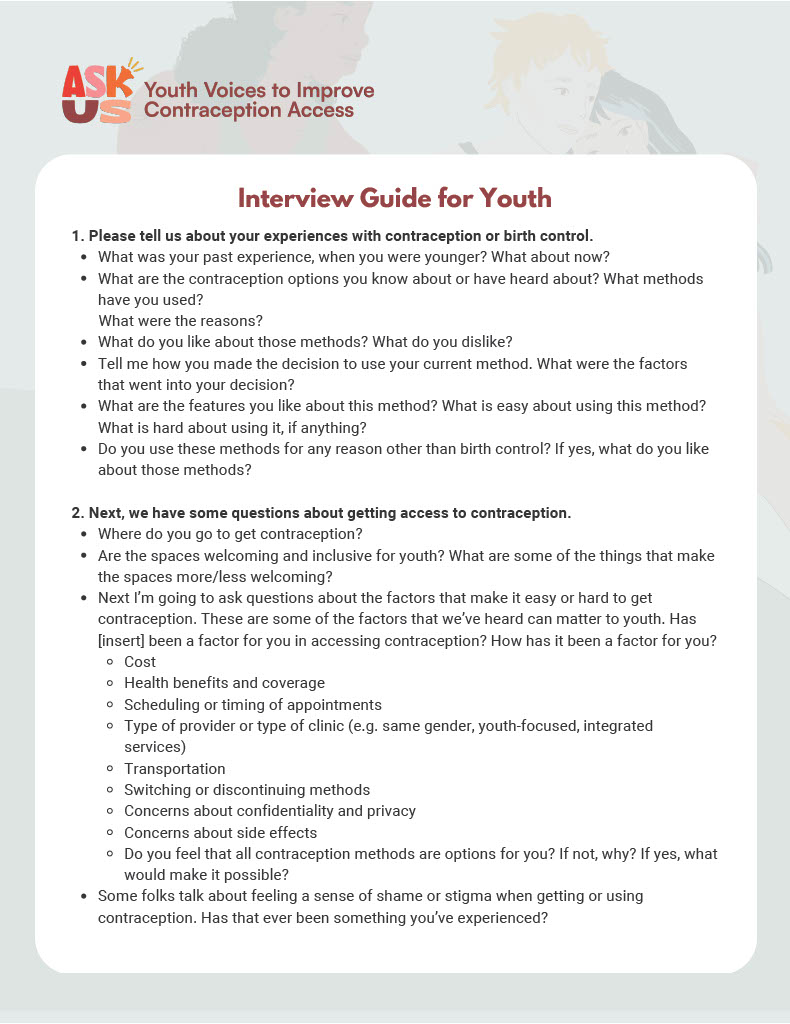

Supplement: Supplement 4. Interview Guides 1 [file ZRHM_A_2678063_SM8662.jpg]

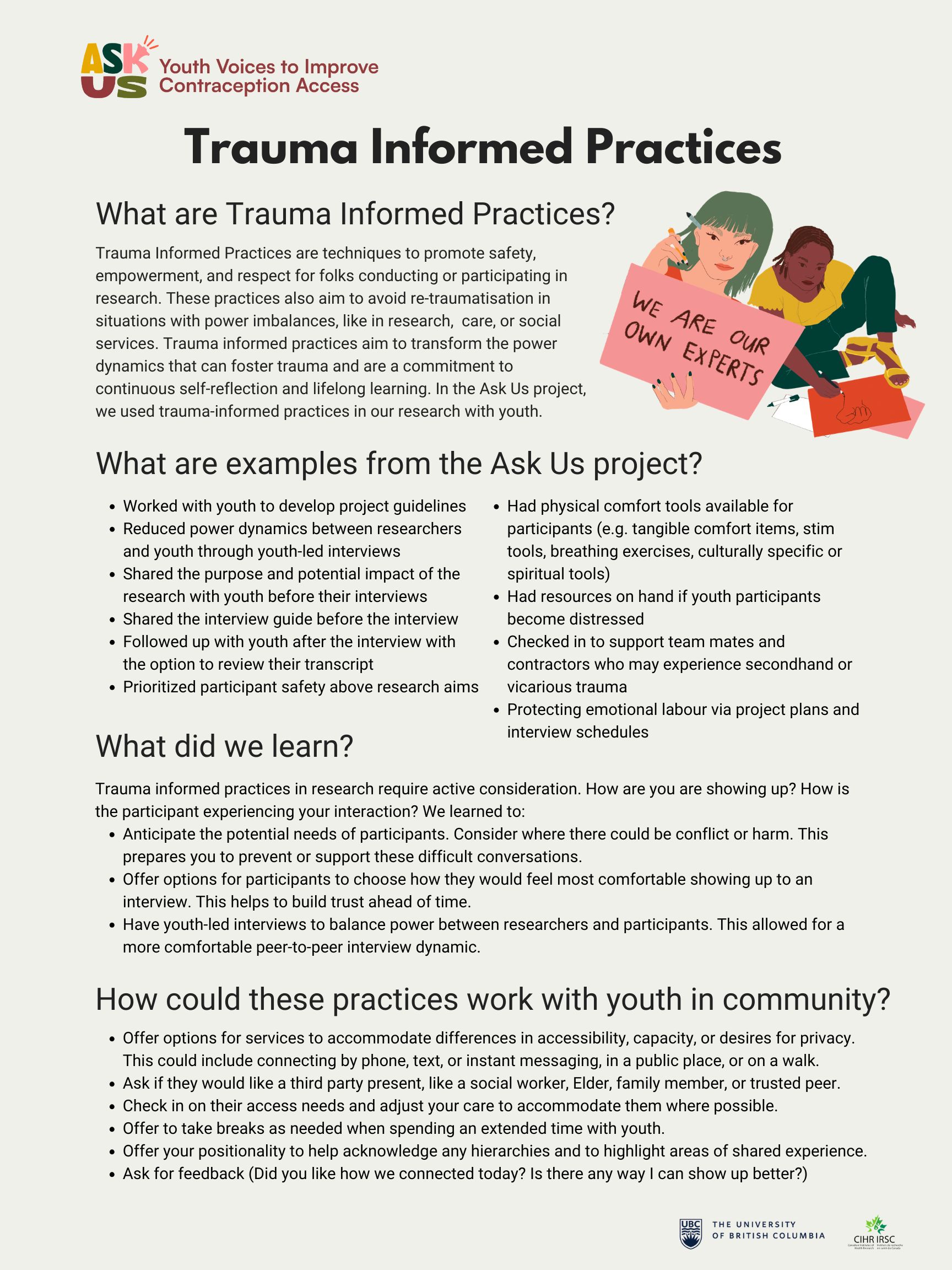

Supplement: Supplement 2. Trauma Informed Guide [file ZRHM_A_2678063_SM8661.jpg]

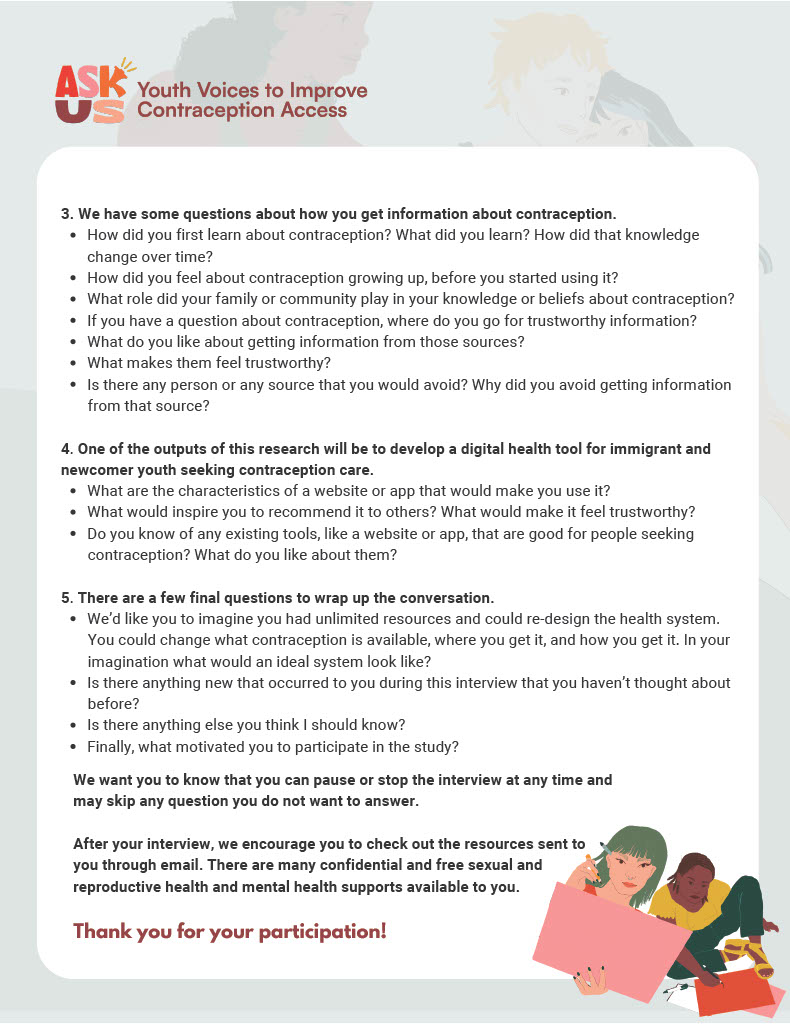

Supplement: Supplement 4. Interview Guides 2 [file ZRHM_A_2678063_SM8660.jpg]

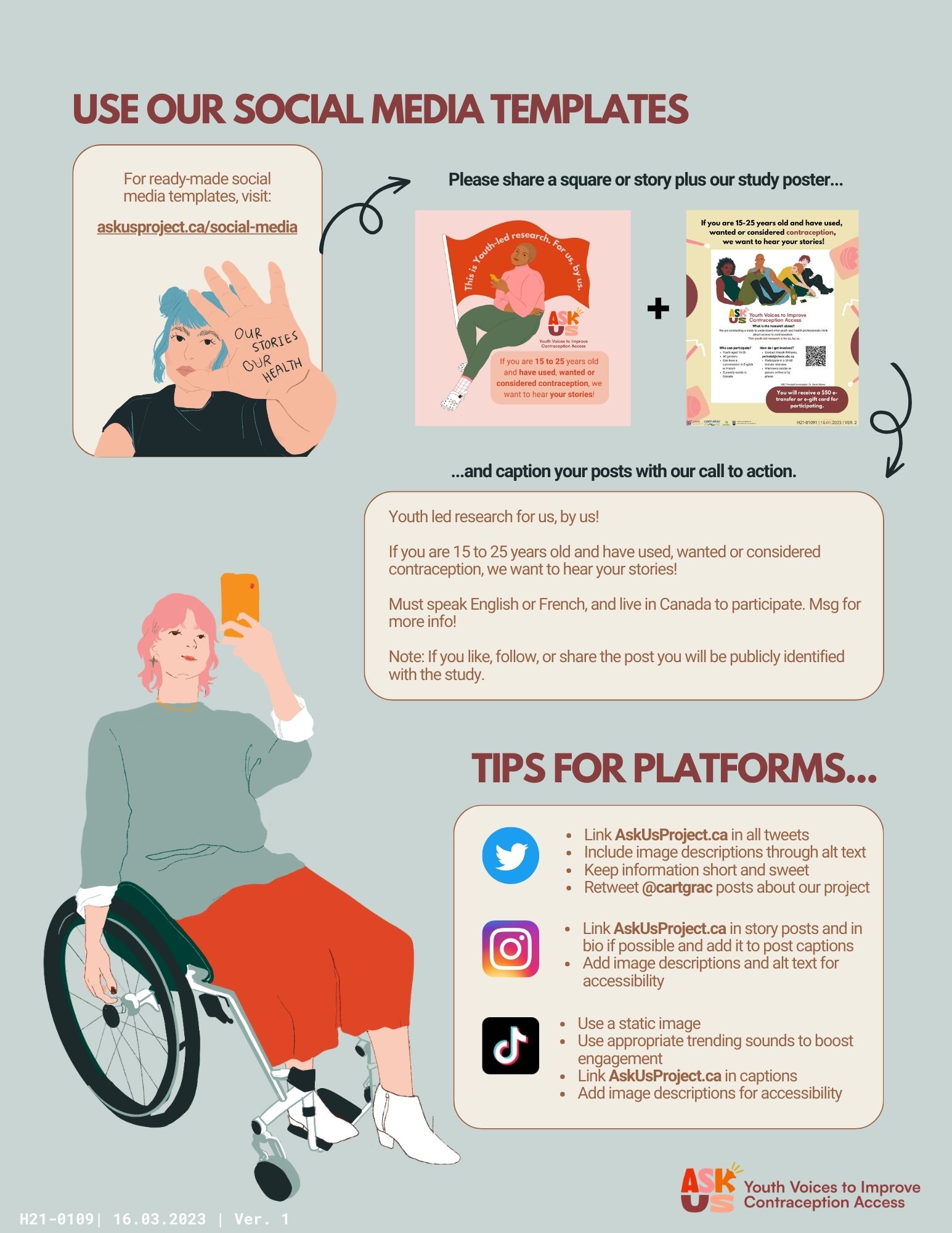

Supplement: Supplement 3. Social Media Guide 2 [file ZRHM_A_2678063_SM8659.jpg]
